# New records for millipedes from southern Chile (Polydesmida: Dalodesmidae; Polyzoniida: Siphonotidae)

**DOI:** 10.3897/BDJ.5.e15919

**Published:** 2017-09-09

**Authors:** Robert Evan Mesibov

**Affiliations:** 1 No affiliation for this profile, West Ulverstone, Tasmania,, Australia

**Keywords:** Polydesmida, Dalodesmidae, Polyzoniida, Siphonotidae

## Abstract

**Background:**

Millipedes from 1983 collections by the author in southern Chile have been identified and registered as specimen lots at the Queen Victoria Museum and Art Gallery (QVMAG) in Launceston, Tasmania.

**New information:**

Collection and specimen data from the new QVMAG specimen lots have been archived in Darwin Core format together with a KML file of occurrences. The 31 occurrence records in the Darwin Core Archive list 13 millipede taxa from 16 sites in Llanquihue and Osorno provinces, Chile.

## Introduction

The millipede fauna of southern South America is poorly known Golovatch 2014, and many named species are known only from their type localities. While working as a forester in 1983 I had the opportunity to visit native forests in southern Chile and to opportunistically sample myriapods. Centipedes in the samples were later deposited in the Tasmanian Museum and Art Gallery (TMAG) in Hobart, Tasmania, and millipedes in the Queen Victoria Museum and Art Gallery (QVMAG) in Launceston, Tasmania. I recently re-examined the QVMAG samples and was able to identify most of the millipedes to species, and to register them as QVMAG specimen lots. Collection and specimen data for these registered lots have now been archived and are publicly available through Zenodo.

## General description

### Purpose

The occurrence data are being made available to assist future studies of the millipede fauna of southern South America. The specimen lots are available for loan from QVMAG.

## Sampling methods

### Sampling description

Millipedes were opportunistically hand-collected by the author during visits to forests in Chile's Llanquihue and Osorno provinces in 1983. Most were found in or under rotting logs. Millipedes were placed in ca 75% methylated spirits in Chile and transferred to 75-80% ethanol at QVMAG. Using my 1983 field notes, Google Maps, Google Earth and online maps of southern Chile, I georeferenced the collecting localities in 2017 to a spatial uncertainty of ±1000 m or less in most cases.

## Geographic coverage

### Description

Llanquihue and Osorno provinces in southern Chile

### Coordinates

-42.960 and -40.160 Latitude; -74.089 and -72.167 Longitude.

## Taxonomic coverage

### Taxa included

**Table:** 

Rank	Scientific Name	
family	Dalodesmidae Cook, 1896	
family	Siphonotidae Cook, 1895	

## Temporal coverage

**Data range:** 1983-4-19 – 1983-6-15.

## Usage rights

### Use license

Other

### IP rights notes

By agreement with QVMAG, the data in the Darwin Core Archive are made available on a Creative Commons Attribution 4.0 licence (http://creativecommons.org/licenses/by/4.0/legalcode)

## Data resources

### Data package title

Chile_Diplopoda_1983_QVMAG.zip

### Resource link

http://doi.org/10.5281/zenodo.886678

### Alternative identifiers

https://zenodo.org/record/886678

### Number of data sets

1

### Data set 1.

#### Data set name

Specimen data for millipedes collected in 1983 in Chile by Robert Mesibov, and held in the Queen Victoria Museum and Art Gallery, Launceston, Tasmania, Australia (Polydesmida: Dalodesmidae; Polyzoniida: Siphonotidae)

#### Data format

Darwin Core Archive

#### Number of columns

38

#### Character set

UTF-8

#### Download URL

https://zenodo.org/record/886678/files/Chile_Diplopoda_1983_QVMAG.zip

#### Description

Archive containing the four Darwin Core files citations.txt, occurrence.txt, rights.txt and meta.xml, and a KML file organised by taxon, with QVMAG registration number and spatial uncertainty as additional information (in pop-up balloon). The file occurrence.txt contains all occurrence and collecting event data.

**Data set 1.** 

Column label	Column description
occurrenceID	An identifier for the Occurrence
institutionCode	The acronym in use by the institution having custody of the objects referred to in the record
collectionCode	The name identifying the collection from which the record was derived
catalogNumber	A unique identifier for the record within the collection
basisOfRecord	The specific nature of the data record
individualCount	The total number of specimens in the registered specimen lot
sex	Number of males and/or females
lifeStage	Number of adults and/or juveniles
preparations	Preservation method for the specimens
recordedBy	Full name of the collector
kingdom	Taxonomic kingdom of the taxon
phylum	Taxonomic phylum of the taxon
class	Taxonomic class of the taxon
order	Taxonomic order of the taxon
family	Taxonomic family of the taxon
genus	Taxonomic genus of the taxon, if known
specificEpithet	Taxonomic species name of the taxon, if known
scientificNameAuthorship	Author and year of the taxon
taxonRank	Taxonomic rank of the taxon
scientificName	Full scientific name of the taxon; genus+species+author, year if known
identifiedBy	Full name of the taxon identifier
dateIdentified	Date in YYYY-MM-DD format when the taxon was identified
identificationRemarks	Notes (in selected cases) on the identification
locality	Location of the collection site in words
decimalLatitude	Latitude of the collection site in decimal degrees to four decimal places
decimalLongitude	Longitude of the collection site in decimal degrees to four decimal places
geodeticDatum	The datum used for decimalLatitude and decimalLongitude
coordinateUncertaintyInMeters	The horizontal distance (in meters) from the given decimalLatitude and decimalLongitude describing the smallest circle containing the whole of the location
eventDate	Collection date in YYYY-MM-DD format
habitat	Notes on the forest around the collection site
samplingProtocol	The method used for collection in the field
continent	The name of the continent in which the location occurs
country	The name of the country in which the location occurs
countryCode	The two-letter ISO abbreviation for the country
stateProvince	The name of the province in which the location occurs
georeferencedBy	The full name of the georeferencer
georeferencedDate	The date in YYYY-MM-DD format on which the collection site was georeferenced
georeferenceSources	The data sources used to georeference the collection site

## Additional information

The 31 occurrence records in the Darwin Core Archive list 13 millipede taxa from 16 sites in southern Chile. Ten of the 13 taxa (24 samples) are named species of Dalodesmidae (Polydesmida), one sample contains a possibly new species in the genus *Tsagonus* Chamberlin, 1957 (Dalodesmidae) and two samples contain only unidentifiable female dalodesmids. The last taxon (six samples) is Siphonotidae (Polyzoniida).

The Dalodesmidae identified to species are: *Abatodesmus velosoi* Demange & Silva, 1971, *Anaulacodesmus bifidus* Golovatch, 2014, *A. carinobtusus* Silvestri, 1903, *A. levissimus* Attems, 1898, *Pleonaraius pachyskeles* Attems, 1898, *Semnosoma eskovi* Golovatch, 2014, *Trienchodesmus gayanus* (Gervais, 1847), *Tsagonus muermo* Chamberlin, 1957, *T. nahuelbutae* Chamberlin, 1957 and *T. osorno* Chamberlin, 1957. A male *Tsagonus* from forest at Lago San Antonio in the south of Chiloé island is close to *T. silvestrii* Demange & Silva, 1976 but may be a new species.

The Siphonotidae are tentatively referred to *Burinia*, following Golovatch 2014, who refers all indigenous Chilean Polyzoniida to that genus.
